# Risk Factors for Postoperative Osteomyelitis among Patients after Bone Fracture: A Matched Case–Control Study

**DOI:** 10.3390/jcm11206072

**Published:** 2022-10-14

**Authors:** Gulnur Slyamova, Arnur Gusmanov, Arman Batpenov, Nurlan Kaliev, Dmitriy Viderman

**Affiliations:** 1 Department of Medicine, School of Medicine, Nazarbayev University, Kerey and Zhanibek Khans Street 5/1, Astana 010000, Kazakhstan; 2 National Scientific Center Named after Academician Batpenov N.D., Abylay Khan Ave 15 a, Astana 010000, Kazakhstan; 3 Department of Biomedical Sciences, School of Medicine, Nazarbayev University, Kerey and Zhanibek Khans Street 5/1, Astana 010000, Kazakhstan

**Keywords:** osteomyelitis, risk factors, postoperative complications, infection, outcomes

## Abstract

The healthcare burden of osteomyelitis is increasing. Postoperative and posttraumatic osteomyelitis account for 80% of all cases of osteomyelitis. The aim of this study was to find risk factors for postoperative osteomyelitis in Kazakhstan. We included 245 patients admitted to the National Scientific Center of Traumatology and Orthopedics from 2018 to 2020. Cases were matched with controls in a 1:4 ratio. Exact matching was performed by gender, ICD-10, and ICD-9 codes. The main variables included socio-demographics, diagnosis at admission, characteristics of fractures, comorbidities, complications, hospitalization milestones, and osteomyelitis characteristics. Descriptive analyses, along with bivariate analysis and multivariate conditional logistic regression, were performed. Open fracture (adjOR = 6.25; 95%CI 1.64–23.79), the presence of complications of initial fracture (adjOR = 3.46, 95%CI 1.13–10.56), comminuted fracture form (adjOR = 1.87; 95%CI 0.73–4.75), a positive history of diabetes or blood glucose >7 g/L (adjOR = 4.25; 95%CI 1.26–14.3), incision or wound length of more than 10 cm (adjOR = 6.53; 95%CI 1.1–38.6), additional implanted medical item (adjOR = 1.23; 95% CI 1.1–1.47), and unemployment or being retired (adjOR = 4.21; 95%CI 1.74–10.18) were found to be significant predictors of postoperative osteomyelitis. Almost all our findings are concordant with previous studies, except for the type of fracture. Different authors report conflicting results on the latter potential risk factor. Therefore, prospective studies on this issue are required.

## 1. Introduction

Osteomyelitis is characterized by bone deterioration induced by bacterial infections [1]. This condition is difficult to diagnose and manage due to the heterogeneity of its pathogenesis and clinical presentation [2]. According to Walter et al. [3], posttraumatic and postoperative osteomyelitis are becoming more common, now accounting for around 80% of all cases of osteomyelitis.

Infectious complications of proximal femoral bone fracture can increase cost of the treatment by over three times [4]. In the case of open fractures, infections increase the cost of treatment and hospitalization by 63% and 80%, respectively [5]. The estimated average duration of hospital stay for osteomyelitis patients is 17.5 days, and 20% of patients are re-admitted to hospital [6]. Chronic osteomyelitis increases morbidity and mortality, especially in the elderly with other comorbidities [7]. For instance, osteomyelitis can increase amputation rates in type 2 diabetes patients with burns to the lower extremities [8].

Previous studies have identified various factors that potentially increase the risk of developing osteomyelitis after surgeries among patients admitted with fractures. Bacterial biofilms attached to orthopedic devices implanted during the operation (*S. aureus* and *S. epidermidis*) can increase the rate of postoperative infection complications [9]. Open fractures are potentially more prone to the development of infectious complications because of the direct intrusion of foreign bacterial flora [10]. The development of disease may be associated with the severity of the fracture and the extent of tissue injury [11,12]. Male sex, advanced age, and diabetes were also associated with an increased risk of osteomyelitis following fracture [13]. These findings are largely rooted in studies investigating military populations. The limitations of previous studies of the general population include using case series design and sampling from the same ethnic and racial populations. There is a lack of well-designed studies on the epidemiology of postoperative osteomyelitis in populations from developing countries.

Considering the restraints of previous studies, we aimed to investigate risk factors for postoperative osteomyelitis in patients admitted for fractures in Kazakhstan. We hypothesized that the factors associated with post-operative osteomyelitis in developing countries might differ from the factors in developed countries.

## 2. Materials and Methods

### 2.1. Patients

We included patients admitted to the National Scientific Center of Traumatology and Orthopedics (NSCTO) in Nur-Sultan city in the period from 2018 to 2020. Age, sex, diagnoses at admission, and provided procedures were retrieved from the Health Information System (HIS) and used to identify cases and controls. The International Classification of Diseases 10th Revision (ICD-10) codes, namely S40–S99, were used to tag these patients.

**Figure 1:**
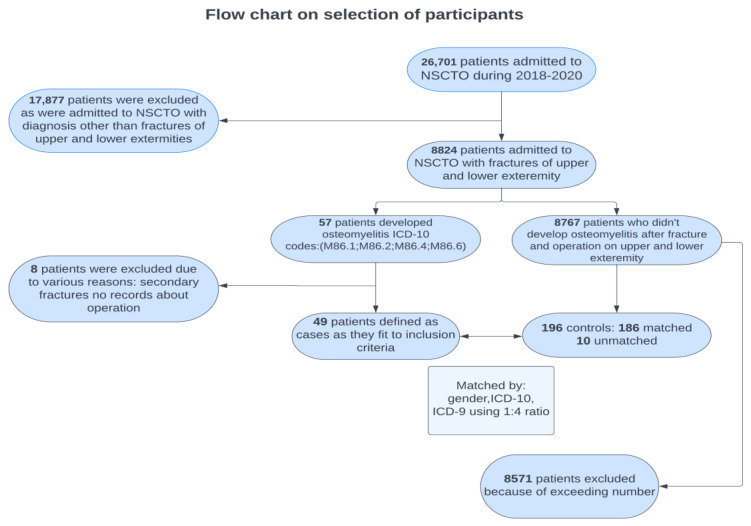
Patient flow chart.

A matched case–control design was used for a given study (Figure 1). Inclusion criteria for cases were (1) being hospitalized with a fracture of the upper and/or lower extremities during 2018–2020 (ICD-10 codes: S40–S99); (2) having a surgical procedure as a treatment for a fracture; (3) being readmitted to the hospital with osteomyelitis; and (4) a causal link between previous fracture and osteomyelitis as identified by a physician. Medical procedures were coded in HIS records using ICD-9-CM Diagnosis and Procedure (ICD-9) codes. Osteomyelitis was identified by ICD-10 codes for other acute osteomyelitis (M86.1), subacute osteomyelitis (M86.2), chronic osteomyelitis with draining sinus (M86.4), and other chronic osteomyelitis (M86.6). The causal link between previous fracture and osteomyelitis was established through medical records, namely the section on the history of current disease described by a physician. Additional criteria included precise indication of the connection between developing osteomyelitis and previous fracture, as well as correct sequence of admissions. Exclusion criteria for cases were: (1) not meeting the inclusion criteria and (2) missing data in medical records.

Cases were matched with controls in a 1:4 ratio. Eligible controls were those who had fracture and surgeries performed in NSCTO during the same years but did not develop osteomyelitis afterwards. A total of 186 patients were matched with cases by sex, fracture location, and surgery. Matching on an initial fracture location was performed by first letter and two subsequent digits in the ICD-10 code (i.e., S42 for fracture of shoulder and upper arm). The first two digits of the ICD-9 codes were used for matching by provided surgical procedure (i.e., 79 for reduction of fracture and dislocation). To meet the 1:4 ratio between cases and controls, ten patients were randomly selected from an initial pool of controls.

### 2.2. Exposure Variables

After the identification of cases and controls, discharge data were collected from HIS for the collection of socio-demographic, health- and trauma-related variables. Patient age was coded in years as a continuous variable. Occupational status was coded as “employed” and “unemployed/retired”. Patient height and weight were used to calculate body mass index (BMI).

Variables related to initial fracture were collected, such as fracture type (“open”, “closed”), fracture location (“upper” and “lower extremities”), days from the moment of fracture and admission, and complications of initial fracture (“No”, “Yes”). Complications of initial fracture included dislocations, hemarthrosis, radial nerve neuropathy, and tendon and syndesmoses injury. Forms of fracture were categorized as comminuted or others, which consisted of obliques, spiral, and transverse forms. Causes of trauma included street injury, domestic trauma, and others, consisting of sports, occupational and criminal trauma. Alcohol intoxication at admission was recorded. Hypertension, chronic vascular diseases, and chronic cardiac diseases were evaluated from medical history. Diabetes mellitus and blood glucose levels higher than 7 mmol/L were identified if there was a corresponding medical record or measurement at admission. Pyelonephritis, cystitis, and presence of bacteria and leukocytes in urinalysis were coded as urinary tract infections. Anemia or erythrocytes (<4*10^12^/L), and hemoglobin (<130 g/L for men and <120 g/L for women) levels were searched for in medical records. Systemic inflammatory response syndrome was defined as leukocytes >9*10^9^/L and sedimentation rate of erythrocytes of >12 mm/h for men and >15 mm/h for women. Duration of operation in minutes, type of anesthesia (“spinal”, “general”, and “others”), length of incision or wound (“0–2 cm”, “3–10 cm”, and “>10 cm”), and the number of implanted medical items were collected. The length of antibiotic treatment in days was analyzed and the treatment was grouped according to the presence of cephalosporins, as they were prescribed to the majority of patients. Additional variables specifically related to the development of osteomyelitis were collected for cases: location of a process, pathogen, and days from fracture until hospitalization with osteomyelitis.

### 2.3. Statistical Analysis

Continuous variables were summarized using mean and standard deviation (SD), while frequencies and relative frequencies were used for categorical variables. In bivariate analysis, to test the association between continuous exposure variables and the outcome of developing osteomyelitis, independent two-sample t-test was used as a first choice and the Mann–Whitney U test was a non-parametric alternative. Similarly, for bivariate analysis between categorical independent variables and outcome, Pearson’s Chi-squared test and Fisher’s exact test were the priority and alternative tests, respectively. Since cases were matched with controls by several characteristics, conditional logistic regression was utilized to build a model that included significant predictors of osteomyelitis development. By adding those variables into single model, it was aimed to eliminate confounding effects. A backward stepwise model selection approach was utilized. Variables with a *p*-value < 0.25 in bivariate analysis were included in a multivariate regression model and non-significant variables (*p* > 0.05) were excluded from the model one by one. Some variables (age, forms of fracture, BMI, days before admission, and post-operative stay-days) remained in the final model despite having non-significant results due to epidemiological importance and being well-recognized confounding variables. Crude and adjusted odds ratios with corresponding 95% confidence intervals (CI) were reported. This study was approved by NUSOM Institutional Research Ethics Committee on 26 November 2021 (IREC number NOV#09).

## 3. Results

### 3.1. Descriptive Analysis

Patient flow is presented in Figure 1. Socio-demographic characteristics of patients are presented in Table 1. There were a total of 56 (22.9%) female and 189 (77.1%) male patients who received surgical procedure as a treatment for fracture; 43.3% of patients were either unemployed or pensioners. The mean number of hospital stay-days was 10.8 (±5.7). Comminuted fracture took place in roughly one-third of patients (38.4%), while the rest experienced other forms of fracture. Overall, 58.8% of patients developed complications after fracture and 28 patients (6.9%) had either prior-diagnosed diabetes or blood glucose level more than 7 g/L. Participants had on average 7.5 (±3.4) implanted medical items.

### 3.2. Bivariate Analysis

The bivariate analysis between potential risk factors and osteomyelitis development is presented in Table 2.

Unemployed or pensioners were more prevalent among cases (69.4%) compared to controls (36.7%). More cases experienced open fractures (22.5%) as well as comminuted fractures (53.1%) compared to controls—11.7% and 34.7%, respectively (*p* = 0.05). Patients with pre-diagnosed diabetes or blood glucose more than 7 g/L were more frequently observed among cases (*p* = 0.02). Cases had significantly more implanted medical items on average compared to controls—7.1 (±3.6) and 5.1 (±3.3) items respectively.

### 3.3. Multivariate Analysis of Risk Factors for Osteomyelitis Development

In conditional multivariate logistic regression analysis, six variables were found to be significant predictors for developing osteomyelitis (Table 3). These were open fractures, complications of initial fracture, history of diabetes or hyperglycemia at admission, incisions or wounds longer than 10 cm, implanted medical items, and unemployed or retired status. The adjusted odds of developing osteomyelitis were higher among those who had open fracture compared to patients with closed fracture (adjOR = 6.25 [95%CI: 1.64–23.79], *p* = 0.007). The presence of complications of initial fracture was significantly associated with an outcome (adjOR = 3.46 [95%CI: 1.13–10.56], *p* = 0.03). Patients with pre-diagnosed diabetes or blood glucose levels of more than 7 g/L were more likely to develop osteomyelitis (adjOR = 4.25 [95%CI: 1.26–14.3], *p* = 0.02). An additional implanted medical item for bone fixation was associated with an increase in the adjusted odds of developing osteomyelitis by 27% (95%CI: 1.1–1.47, *p* = 0.001). Unemployed or pensioner status resulted in higher odds of developing the outcome compared to employed patients, after controlling for other covariates (adjOR = 4.21 [95%CI: 1.74–10.18], *p* = 0.001).

## 4. Discussion

We examined risk factors of osteomyelitis in patients with fractures of the upper and lower extremities. Our findings are consistent with the results of studies that have associated a history of diabetes or stress-induced hyperglycemia with infectious complications including osteomyelitis [13,14,15]. Patients with diabetes tend to have impaired bone regeneration [16,17]. A recent study reported the dysfunction of angiocrine signaling from pericytes in bone marrow of diabetic patients [18]. This leads to impaired blood circulation at the fracture site. Since diabetic patients have bone fragility and a consequent predisposition for fractures, the development of detailed algorithms for assessing the risk of having osteomyelitis in this population is of great importance.

Our results are comparable to the studies reporting higher susceptibility of patients with foreign body implants to infection [9,10]. Implant-related infections occur due to bacterial adhesion to the surface of an implant and the subsequent biofilm formation. Bacterial invasion starts a chain of biochemical processes that results in bone necrosis and osteolysis [19]. Current strategies combating biofilm formation include coating with antimicrobial agents, antibiofilm vaccines, and using inhibitors of bacterial adhesion [20,21]. However, there is a still insufficient knowledge of the efficacy and safety of the mentioned strategies.

We also found that type of the fracture (open or close) is an independent risk factor for osteomyelitis (OR 6.25 CI 1.64–23.79). This contradicts the results of the study by Grigorian et al. [22]. However, earlier studies showed results similar to ours [23,24]. More prospective studies are needed to assess the relationship between the type of long bone fractures and acute or chronic osteomyelitis. Open fractures prolong the time required for bone healing and the resulting open wound facilitates the invasion of tissues by causative pathogens of osteomyelitis. Apart from open fractures, comminuted fractures and fractures of the lower extremities were previously found to be significant predictors of osteomyelitis development [25]. Since our current study identified open fractures to be independently associated with osteomyelitis, further studies are required to evaluate the association between other trauma-related characteristics and osteomyelitis.

Our findings suggest that incisions longer than 10 cm result in predisposition to osteomyelitis. This might have been due to the prolonged recovery time of the wound. However, it is confounded by the complexity of the fracture, as compound fractures require more extensive surgical access, thus increasing the length of the incision. Patients with complications of the initial fracture, such as dislocations or ligament rupture and hemarthrosis, have a higher likelihood of developing infectious complications. This finding might be explained by severe disturbance of blood circulation and innervation, consequently leading to ischemia and osteonecrosis. In some cases, fractures accompanied by dislocations might cause joint instability and osteoarthritis and require longer rehabilitation and a delay in postoperative activation.

Unemployed and retired patients had higher odds of developing postoperative osteomyelitis compared to employed individuals in this study. This finding is in accordance with previous investigations [13,26]. Low health status, malnutrition, substandard living conditions, and lack of access to proper health care are important factors that possibly explain the observed association. Retired individuals tend to develop osteomyelitis due to various preexisting disorders that hinder recovery after fracture.

Access to original medical records is a key strength of our study that has reduced recall bias. A 1:4 ratio for cases and controls contributed to the power of the study. This is the first study in Central Asia that investigated the risk factors of postoperative osteomyelitis after bone fracture.

There are a few significant limitations to this study. We believe that having ten unmatched controls may have distorted the results of the study. Another limitation is missing data. As the data in medical records were not designed for the present study, incomplete records were observed. Additionally, since this was a single-center study, the results may not be generalizable to a wider population.

## 5. Conclusions

Our study reports the determinants of acute and chronic osteomyelitis in patients after fracture who underwent surgeries. This is the first case–control study in the Central Asian region that investigates the epidemiology of postoperative osteomyelitis in developing countries. Six independent risk factors were identified, including fracture type, complications of initial fracture, history of diabetes or blood glucose of >7 g/L, incision/wound length of more than 10 cm, number of implanted medical items, and occupation status. Of these factors, incision length and number of implanted medical items were true modifiable factors. Better preoperative risk assessment can identify the group of patients that require additional prophylactic measures.

## Figures and Tables

**Table 1:** Descriptive analysis of socio-demographics and variables related to initial trauma. Ceph, cephalosporins; BMI, body mass index; SD, standard deviation.

Socio-Demographics and Variables at Initial Trauma	Total, *n* = 245
Age, mean ± SD	43.2 ± 14.5
Gender, *n* (%)	
Males	189 (77.1%)
Females	56 (22.9%)
Occupation status, *n* (%)	
Employed	139 (59.7%)
Unemployed/retired	106 (43.3%)
Hospital stay-days, mean ± SD	10.8 ± 5.7
Days before admission, mean ± SD	2.4 ± 5.5
Fracture location, *n* (%)	
Upper extremities	75 (30.6%)
Lower extremities	170 (69.4%)
Fracture type, *n* (%)	
Open	34 (13.9%)
Closed	211 (86.1%)
Complications of initial fracture, Yes, *n* (%)	144 (58.8%)
Forms of fracture, *n* (%)	
Comminuted	94 (38.4%)
Others (oblique, spiral, transverse)	151 (61.6%)
Hypertension, Yes, *n* (%)	33 (13.5%)
History of diabetes or blood glucose > 7 g/L, Yes, *n* (%)	28 (6.9%)
Urinary tract infection, Yes, *n* (%)	48 (19.6%)
Chronic cardiac disease, Yes, *n* (%)	17 (6.9%)
Anemia, Yes, *n* (%)	70 (28.6%)
Vascular disease, Yes, *n* (%)	8 (3.3%)
Coagulopathy, Yes, *n* (%)	87 (38%)
Systemic inflammatory response syndrome, Yes, *n* (%)	194 (79.5%)
Alcohol intake at admission, Yes, *n* (%)	28 (11.4%)
Cause of trauma, *n* (%)	
Street injury	123 (50.2%)
Domestic trauma	76 (31.0%)
Others (Sports, work, criminal trauma)	46 (18.8%)
Type of anesthesia, *n* (%)	
Spinal	155 (63.3%)
General	56 (22.9%)
Others	34 (13.9%)
Operation duration in minutes, mean ± SD	83.5 ± 41.8
Incision/wound length in cm, *n* (%)	
0–2 cm	64 (26.1%)
3–10 cm	151 (61.6%)
>10 cm	30 (12.2%)
# of implanted medical items, mean ± SD	5.5 ± 3.4
Postoperative stay-days, mean ± SD	7.5 ± 4.6
BMI categories, *n* (%)	
Underweight/normal	90 (39.0%)
Overweight	98 (42.4%)
Obese	43 (18.6%)
Length of antibiotic therapy, mean ± SD	5.6 ± 5.3
Antibiotic treatment, *n* (%)	
Ceph	190 (77.6%)
Ceph + other antibiotic groups	34 (13.9%)
No	14 (5.7%)
Other antibiotic groups	7 (2.9%)

**Table 2:** Bivariate analysis of sociodemographics and variables related to initial trauma with osteomyelitis.

Socio-Demographics and Variables at Initial Trauma	Patients Who Did Not Develop Osteomyelitis (*n* = 196)	Patients Who Developed Osteomyelitis (*n* = 49)	*p*-Value
**Age**, mean ± SD	42.7 ± 1.0	45.2 ± 2.2	0.28 *
**Gender**, *n* (%)			0.65 **
Males	150 (76.5%)	39 (79.6%)	
Females	46 (23.5%)	10 (20.4%)	
**Occupation status**, *n* (%)			<0.001 **
Employed	124 (63.3%)	15 (30.6%)	
Unemployed/retired	72 (36.7%)	34 (69.4%)	
**Hospital stay-days**, mean ± SD	10.8 ± 5.6	12.2 ± 5.8	0.06 *
**Days before admission**, mean ± SD	2.5 ± 5.9	2.0 ± 3.6	0.6 *
**Fracture location**, *n* (%)			0.73 **
Upper extremities	59 (30.1%)	16 (32.7%)	
Lower extremities	137 (69.9%)	33 (67.4%)	
**Fracture type**, *n* (%)			0.05 **
Open	23 (11.7%)	11 (22.5%)	
Closed	173 (88.3%)	38 (77.5%)	
**Complications of initial fracture**, *n* (%)			0.09 **
Yes	110 (56.1%)	34 (69.4%)	
No	86 (43.9%)	15 (30.6.%)	
**Forms of fracture**, *n* (%)			0.02 **
Comminuted	68 (34.7%)	26 (53.1%)	
Others (oblique, transverse, trimalleolar, etc.)	128 (65.3%)	23 (46.9%)	
**Hypertension**, Yes, *n* (%)	24 (12.2%)	9 (18.4%)	0.26 **
**History of diabetes or blood glucose > 7 g/L**, Yes, *n* (%)	10 (5.1%)	7 (14.3%)	0.02 **
**Urinary tract infection**, Yes, *n* (%)	35 (17.9%)	13 (26.5%)	0.17 **
**Chronic cardiac disease**, Yes, *n* (%)	12 (6.1%)	5 (10.2%)	0.32 **
**Anemia**, Yes, *n* (%)	57 (29.1%)	13 (26.5%)	0.72 **
**Coagulopathy**, Yes, *n* (%)	71 (39.4%)	16 (32.7%)	0.39 **
**Systemic inflammatory response syndrome**, Yes, *n* (%)	150 (76.9%)	44 (89.8%)	0.05 **
**Alcohol at admission**, Yes, *n* (%)	20 (10.2%)	8 (16.3%)	0.23 **
**Cause of trauma**, *n* (%)			0.86 **
Street injury	97 (49.5%)	26 (53.1%)	
Domestic trauma	61 (31.1%)	15 (30.6%)	
Others (Sports, work, criminal trauma)	38 (19.4%)	8 (6.3%)	
**Type of anesthesia**, *n* (%)			0.81 **
Spinal	124 (63.3%)	31 (63.3%)	
General	46 (23.5%)	10 (20.4%)	
Others	26 (13.3%)	8 (16.3%)	
**Operation duration in minutes**, mean ± SD	81.3 ± 41.0	92.3 ± 44.4	0.1 *
**Incision/wound length in cm**, *n* (%)			0.15 **
0–2 cm	53 (27.0%)	11 (22.5%)	
3–10 cm	123 (62.76%)	28 (57.1%)	
>10 cm	20 (10.2%)	10 (20.45)	
**# of implanted medical items**, mean ± SD	5.1 ± 3.3	7.1 ± 3.6	<0.001 *
**Postoperative stay-days**, mean ± SD	7.4 ± 4.5	7.6 ± 4.6	0.81 *
**BMI categories**, *n* (%)			0.17 **
Normal	71 (38.8%)	19 (39.6%)	
Overweight	82 (44.8%)	16 (33.3%)	
Obesity	30 (16.4%)	13 (27.1%)	
**Length of antibiotic therapy**, mean ± SD	5.3 ± 5.1	6.5 ± 6.1	0.17 *
**Antibiotic treatment**, *n* (%)			0.68 **
Ceph	155 (79.1%)	35 (71.4%)	
Ceph + other antibiotic groups	26 (13.3%)	8 (16.3%)	
No	10 (5.1%)	4 (8.2%)	
Other antibiotic groups	5 (2.5%)	2 (4.1%)	

* Ceph, cephalosporins; BMI, body mass index; SD, standard deviation. * Independent two sample *t*-test, ** Pearson’s Chi-squared test.

**Table 3:** Bivariate and multivariate logistic regression analyses of association between socio-demographics and variables related to trauma with osteomyelitis.

Variables	Crude Odds Ratio (95%CI)	*p*-Value	Adjusted Odds Ratio * (95%CI)	*p*-Value
Age	1.02 (1.0–1.05)	0.08	0.99 (0.96–1.03)	0.58
Fracture type		0.06		0.007
Closed	Ref.		Ref.	
Open	2.36 (0.96–5.81)		6.25 (1.64–23.79)	
Complications of initial fracture		0.01		0.03
No	Ref.		Ref	
Yes	3.35 (1.33–8.41)		3.46 (1.13–10.56)	
Forms of fracture		0.009		0.19
Others (oblique, spiral, transverse)	Ref.		Ref	
Comminuted	2.48 (1.26–4.88)		1.87 (0.73–4.75)	
History of diabetes or blood glucose > 7 g/L		0.02		0.02
No	Ref.		Ref	
Yes	3.01 (1.21–7.45)		4.25 (1.26–14.3)	
Incision/wound length in cm				
<3 cm	Ref.		Ref	
3–10 cm	1.6 (0.56–4.54)	0.38	2.37 (0.62–9.01)	0.21
>10 cm	4.38 (1.15–16.65)	0.03	6.53 (1.1–38.6)	0.04
# of implanted medical items	1.23 (1.11–1.37)	<0.001	1.27 (1.1–1.47)	0.001
BMI categories				
Normal	Ref.		Ref	
Overweight	0.75 (0.36–1.55)	0.43	0.87 (0.36–2.1)	0.76
Obese	1.46 (0.61–3.5)	0.4	1.12 (0.34–3.68)	0.85
Occupation status		<0.001		0.001
Employed	Ref.		Ref	
Unemployed/retired	3.9 (1.99–7.65)		4.21 (1.74–10.18)	
Days before admission	0.98 (0.92–1.05)	0.59	1.02 (0.93–1.11)	0.69
Postoperative stay-days	1.01 (0.94–1.08)	0.81	0.9 (0.8–1.02)	0.09

BMI, body mass index; 95%CI, 95% confidence interval; Ref, reference group. * Multivariable logistic regression model includes all variables presented in this table.

## Data Availability

Not applicable.
